# The Sukaribit Smartphone App for Better Self-Management of Type 2 Diabetes: Randomized Controlled Feasibility Study

**DOI:** 10.2196/46222

**Published:** 2024-01-10

**Authors:** Cecilia Josefsson, Thea Liljeroos, Margareta Hellgren, Ulrika Pöder, Mariann Hedström, Erik M G Olsson

**Affiliations:** 1 Department of Public Health and Caring Sciences Uppsala University Uppsala Sweden; 2 Department of Women's and Children's Health Uppsala University Uppsala Sweden; 3 Skaraborg Institute Skövde Sweden

**Keywords:** diabetes mellitus, type 2, health behavior, mobile health, mobile application, pilot study, mobile app, mHealth, diabetes, diabetic, RCT, randomized, glycemic, self care, self management, blood sugar, T2D, diabetes type 2, home-testing, digital health

## Abstract

**Background:**

A new app, Sukaribit, was designed to enable contact between the caregiver and the patient with the intent to improve self-care and glycemic control (hemoglobin A_1c_ [HbA_1c_]).

**Objective:**

This study investigated the feasibility of the study methodology and the intervention in preparation for a larger effectiveness study.

**Methods:**

Adults with type 2 diabetes were recruited in this randomized controlled feasibility study with a mixed methods design. The intervention group (n=28) tried Sukaribit for 2 months. They were encouraged to report blood glucose levels and medications, and they received feedback from a physician. The control group (n=31) received standard care. Both groups were evaluated with pre and postmeasurements of glycemic control (HbA_1c_), diabetes distress, physical activity, and self-care. Feasibility was evaluated against 5 progression criteria regarding recruitment, study methods, and active participation.

**Results:**

Of the 5 progression criteria, only 2 were met or partially met. The recruitment process exceeded expectations, and data collection worked well for self-reported data but not for HbA_1c_ measured with a home testing kit. The participants were less active than anticipated, and the effect sizes were small. Only the number of blood glucose tests per day was positively affected by the intervention, with 0.6 more tests per day in the intervention group.

**Conclusions:**

Recruitment of participants to a future fully powered study may work with minor adjustments. The collection of HbA_1c_ using home testing constituted a major problem, and an alternative strategy is warranted. Finally, the app was not used as intended. In order to proceed with a larger study, the app and study procedures need improvement.

## Introduction

### Background

Type 2 diabetes is a serious disease affecting the prognosis of many other diseases, including cardiovascular disease. Diabetes increases the risk of acute myocardial infarction, stroke, and heart failure [1,2]. To reduce the risk of both microvascular and macrovascular complications, it is important to control blood glucose levels [3] (ie, glycemic control), blood pressure, and lipid levels [4]. For people with type 2 diabetes, a prerequisite for good glycemic control is regular and frequent self-monitoring and knowledge of how blood glucose levels respond to food and physical activity. Many patients have elevated levels of blood glucose, which suggests that self-management is often suboptimal [5].

### Diabetes and Mobile Apps

Even if technical solutions to support diabetes self-management, such as smartphone apps, have become more common, they are used by a minority of patients [6]. It is not clear how many people use diabetes apps in Sweden, but in Australia, only 8% of people reportedly use diabetes mobile apps [6], despite almost unlimited availability with thousands of apps on the market. Reasons for people with diabetes to not use apps can be unawareness of their existence, technical literacy barriers, no need (the disease is not that bad or self-management is sufficient anyway), no recommendation from a health care professional, the resulting increased accountability for one’s own behaviors, or the time-consuming nature of some apps [7,8]. However, studies have shown that people with type 2 diabetes want to use smartphone apps, to reduce not only the practical burden but also the cognitive and emotional burden of diabetes self-management [9]. Studies also have shown that patients want to have more contact with their nurse or physician through digital media than is the case today [9,10]. The most effective app-based technical solutions, in terms of the potential to reduce hemoglobin A_1c_ (HbA_1c_), are interactive and include components such as patient-generated health data, individualized feedback, 2-way communication, and tailored education [11,12]. These components are in line with the 2 behavior change techniques of “feedback on behavior” and “self-monitoring of behavior” that are associated with better glycemic control [13].

### Sukaribit Smartphone App

**Figure 1:**
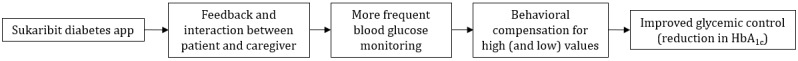
How the diabetes app is intended to improve glycemic control.

The smartphone app Sukaribit (Beta version 1.1, Maishabit AB) was developed with a special focus on the interaction between the patient and caregiver. It has an intentionally basic design to be usable with more basic mobile phones, as it needs less capacity. The app stores and displays blood glucose measurements (patient-generated health data), enables digital 2-way patient-physician or nurse communication, provides individualized feedback, and delivers tailored education. For example, if the person with diabetes enters blood glucose measurements or steps (self-monitoring of behavior), the clinician can provide individualized feedback via the 2-way communication mechanism. The physician can give advice about medications or empower health-related behaviors (feedback on behaviors). The app aims to result in more frequent measurements, better blood glucose control, and better self-efficacy, which could be reflected in more optimal HbA_1c_ (see Figure 1). Sukaribit aims to complement standard care by enabling feedback from the caregiver when patients are not at the clinic. There are several diabetes apps on the market. However, the American Diabetes Association requests longer-term clinical evidence, and clinical outcomes have been published in peer-reviewed literature for only a few diabetes smartphone apps [14]. In line with the British Medical Research Council guidelines for developing and evaluating complex interventions [15], this is the first scientific evaluation of the feasibility of the diabetes app Sukaribit.

### Aim

The purpose of this study was to investigate the feasibility of the study methodology and the intervention before conducting a larger effectiveness study. Our research questions were as follows: (1) Are the study procedures feasible and effective? (2) Is the Sukaribit smartphone app (version 1.1) usable and accepted by people with type 2 diabetes? (3) How large are the effect sizes for the use of the Sukaribit smartphone app on HbA_1c_ and other potential outcomes? In line with recommendations for feasibility evaluations, we developed predetermined progression criteria to decide whether to proceed to a full-scale randomized trial [16].

## Methods

### Research Design

The study was a randomized feasibility study with pre and postmeasurements from an intervention group and a control group. The control group received standard care. The report follows the Consolidated Standards of Reporting Trials (CONSORT) guidelines [17].

### Ethical Considerations

The trial protocol was approved by the Swedish ethical review authority (diary number 2020-04894), and the participants provided written informed consent.

### Progression Criteria

The aim was to study (1) the feasibility of study procedures and (2) the usability and acceptability of the intervention. This follows general recommendations for pilot studies by Avery et al [16]. In addition, we also studied (3) preliminary effect sizes (see the Preliminary Effect Sizes section). Aims (1) and (2) were evaluated against predetermined progression criteria (see Textbox 1) [16]. These progression criteria were set prospectively by the authors considering the possibility of finalizing recruitment of participants for a fully powered randomized controlled trial (RCT) within approximately 2 years and having an activity level in the intervention high enough to draw conclusions about its use. If the progression criteria were met, this indicated that a larger study is feasible using the procedure evaluated; otherwise, revisions should be considered.

**Research questions 1 and 2 and their respective progression criteria.**

(1) Are the study procedures feasible and effective?

1. At least 60 people reported interest in participating in the study within 3 months of recruitment.
2. At least 50% of those who reported interest were eligible for inclusion in the study (ie, met the inclusion but not the exclusion criteria).
3. At least 75% of those randomized (to any of the groups) in the study completed the postmeasurements (ie, had complete data).

(2) Is the Sukaribit smartphone app usable and accepted by patients with type 2 diabetes?

1. At least 80% of those initially interested and eligible actually started participating.
2. At least 50% of those who participated in the intervention sent at least 8 blood glucose measurements during the 2 months the intervention lasted (about 1 per week).

Feasibility data were collected in a log by the research assistant, and the automated activity log from the Sukaribit app was shared with the researchers by Maishabit AB. To further explore if the app is usable and accepted by people with type 2 diabetes, an additional qualitative evaluation was conducted. Participants were asked open-ended questions in the portal about opinions and possible improvement of the app. The intervention group also participated in semistructured telephone interviews for further input about the acceptability of the intervention. The interview guide contained questions about the participant’s diabetes, self-care, and study participation, as well as about the mobile app. The interviews were audio-recorded (average length: 25 minutes) and transcribed. The physician was also interviewed about participation with a separate but similar interview guide.

### Participants and Procedures

The study included 59 adults (age >18 years) with type 2 diabetes. Exclusion criteria were other serious illnesses, HbA_1c_ >70 mmol/mol, BMI <25 kg/m^2^, no regular access to the internet, and not owning a blood glucose monitor. The following 2 initial exclusion criteria were abandoned as they were not that important and not feasible for effective recruitment: people with HbA_1c_ <50 mmol/mol (4 were initially excluded) and an age >65 years.

Participants were recruited (between February 2021 and April 2021) at health care centers in Uppsala, through nationwide adverts in 3 major Swedish newspapers, and via advertising on the national Swedish Diabetes Federation’s web page and in diabetes-specific social media groups. People with type 2 diabetes reported their interest on a study-specific website hosted by Uppsala University or directly to the research assistant via email or telephone. Thereafter, they were contacted by the research assistant who informed them about the study. People who were still interested provided written consent to participate. Thereafter, the research assistant checked the inclusion and exclusion criteria preliminarily and ensured that the participant had a pedometer app on their smartphone or helped them install one.

Participants were sent a home testing kit for HbA_1c_, which meant that they took a blood sample at home and sent it to an accredited laboratory for analysis. As recruitment proceeded, the authors recognized that the wait time for baseline HbA_1c_ test results could be long (mean 15.6, SD 6.4 days). Therefore, we decided to include and randomize participants before the HbA_1c_ test results arrived and exclude them afterwards if necessary; 4 participants were excluded on this premise.

All questionnaires were administered using the Uppsala University Psychosocial Care Program (U-CARE) Portal (the portal). The participants answered the questionnaires at the time of randomization and 8 weeks later (a delayed response of a maximum of 18 days was allowed). Randomization occurred in the portal (see the following paragraph), was totally automated, and occurred in blocks of 6 immediately after the completion of baseline questionnaires.

Those randomized to the intervention were supported in downloading the Sukaribit smartphone app and had a brief user education via telephone. They also received instructions in a PDF brochure. The intervention group was asked to share their blood glucose measurements in the app. Additional follow-up support was requested by 5 participants, as they were uncertain of particular features of the app (eg, input of medications). Those randomized to the control group received standard care [18]. All participants were contacted 2 months later for follow-up data. Those who participated in the intervention were also asked to participate in a semistructured interview about their experience with the intervention. Of those invited to the interview, 16 participants accepted (3 people declined) and were interviewed via telephone by 1 of the 2 research assistants.

### Preliminary Effect Sizes

In addition to the feasibility of the app, the preliminary effect sizes of the Sukaribit smartphone app were also explored. They could be used to calculate the sample size for a fully powered study. Effect sizes were studied for (1) HbA_1c_, (2) number of blood glucose measurements reported the previous week, (3) physical activity, (4) general self-rated health (visual analogue scale from the EQ-5D) [19], (5) diabetes self-management, and (6) diabetes-related distress. This study only explored the changes in these measures, as the study was not sufficiently powered to detect efficacy.

HbA_1c_ was analyzed from a home testing blood test at an accredited laboratory. The blood glucose measurements were recorded by the participants in their own diary of choice and reported in the portal as an outcome. The intervention group could use the app to record their measurements. Physical activity was measured as steps via pedometers on the participants’ smartphones, and the last 7 days were reported in the portal. Participants also reported the number of occasions per week over the last month they had exercised more than 30 minutes for fitness purposes. Diabetes self-management was measured using the Diabetes Self-Management Questionnaire (DSMQ) [20,21]. The DSMQ has 16 items divided into 4 subscales, namely (1) glucose management, (2) dietary control, (3) physical activity, and (4) health care use, with a maximum score of 64. A higher score indicates higher frequency of diabetes self-care behaviors. DSMQ has shown good psychometric properties in several contexts [21]. The Diabetes Distress Scale (DDS) was used to measure diabetes distress [22]. The DDS has 17 items divided into 4 subscales, namely (1) emotional burden, (2) physician-related distress, (3) regimen-related distress, and (4) diabetes-related interpersonal distress, with a maximum score of 102. A higher score indicates more distress. DDS has shown good psychometric properties in several contexts [22].

### The Intervention

The intervention group used the smartphone app Sukaribit (version 1.1) for 2 months. In this app, participants entered their medication list, blood glucose levels, and (optionally) blood pressure levels. Participants could choose to send the recorded measurements to the study physician or not. They were encouraged to send blood glucose measurements at least once a week. The physician was a specialist in family medicine and an associate professor in general practice. She actively participated in the design of the study and evaluated and proposed changes to the app. When measurements were sent, the physician responded with feedback to the participant. All communication occurred through the Sukaribit app. The physician encouraged participants who did not send measurements on their own initiative to register and provide the requested information. This was done at least once for each participant at the start of intervention and regularly approximately once a week if no measurement was sent by the participant during that time. The physician checked messages and measures once a week and replied. There were 2 versions of the app: one for Android and one for iOS.

### Data Analysis

The collected data on recruitment and intervention use were compared with the prespecified progression criteria to decide if they matched. Qualitative data were analyzed with quantitative content analysis [23]. Data from both the interviews and open-ended questions were analyzed together. Within and between-group effect sizes (Cohen *d*) were calculated for HbA_1c_ and self-reported outcomes, dividing the mean differences with pooled SDs, with the aim of being the basis for statistical power and sample size calculations for a future study. The between-group effect sizes used the pooled baseline SDs as recommended by Morris [24]. A value of *d*>0.8 is classified as a large effect size, *d*=0.5 is classified as a medium effect size, and *d*=0.2 is classified as a small effect size according to Cohen [25]. Preliminary inference statistics were also performed utilizing linear regression analysis with the posttreatment value as the outcome and group allocation, baseline values, sex, and age included as covariates. The adjusted estimate can be interpreted as the adjusted mean difference for the treatment group when compared with the control group (the reference). P<.05 was considered significant.

## Results

### Participant Characteristics

Among the randomized participants (n=59), the majority were male (42/59, 71%), born in Sweden (54/49, 92%), and retired (32/59, 54%). The mean age was 61.1 (SD 10.3) years. Most participants (35/59, 59%) reported being lightly active at baseline (eg, practicing yoga, walking, and gardening), with main health issues including hypertension (39/59, 66%) and dyslipidemia (7/59, 46%). Diabetes complications, including eye disease, neuropathy, kidney disease, or sexual dysfunction, were reported by 29% (17/59). For a complete description of the participant characteristics, see Table 1.

**Table 1:** Participant characteristics at baseline (n=59).

Characteristics		Total sample	Treatment	Control
Participants randomized		—^a^	28 (48)	31 (53)
**Sex, n (%)**				
	Female	17 (29)	11 (39)	6 (19)
Age (years), mean (SD)		61.1 (10)	60.2 (12)	61.8 (9)
**Marital status, n (%)**				
	Single	15 (25)	7 (25)	8 (26)
	Cohabiting/married	41 (70)	20 (71)	21 (68)
	Living alone but have a steady partner	3 (5)	1 (4)	2 (7)
	Other	0	0	0
**Country of birth, n (%)**				
	Sweden	54 (92)	25 (89)	29 (94)
	Outside Sweden	5 (9)	3 (11)	2 (7)
**Education level, n (%)**				
	Primary	8 (14)	1 (4)	7 (23)
	Secondary	17 (29)	10 (36)	7 (23)
	University (≤3 years)	17 (29)	7 (25)	10 (32)
	University (>3 years)	17 (29)	10 (36)	7 (23)
**Employment status, n (%)**				
	Student	0	0	0
	Unemployed	2 (3)	0	2 (7)
	Retired	32 (54)	14 (50)	18 (58)
	Employed (any status)	25 (42)	14 (50)	11 (36)
	Employed full time	23 (39)	12 (43)	11 (36)
	Employed part time	2 (7)	2 (7)	0
	Other	0	0	0
**Exercise intensity, n (%)**				
	Mostly sedentary	13 (22)	7 (25)	6 (19)
	Lightly active	35 (59)	17 (60)	18 (58)
	Moderately active	8 (14)	3 (10)	5 (16)
	Very active	3 (5)	1 (4)	2 (6)
Days per week with ≥30 minutes of physical activity, mean (SD)		2.5 (2)	2.3 (2)	2.7 (2)
Steps per day in the last week, mean (SD)^b^		4966 (3862)	4798 (3164)	5094.5 (4371)
Current smoker (Yes), n (%)		6 (10)	2 (7)	4 (13)
**Medical history, n (%)^c^**				
	Hypertension	39 (66)	16 (57)	23 (74)
	Dyslipidemia	27 (46)	11 (39)	16 (52)
	Stroke	1 (2)	0	1 (3)
	History of mental illness	8 (14)	3 (11)	5 (16)
	Myocardial infarction	4 (7)	2 (7)	2 (7)
	Other cardiovascular disease	8 (14)	6 (21)	2 (7)
Diabetes complications (Yes^d^), n (%)^c^		17 (29)	4 (14)	13 (42)
Blood glucose tests per week, mean (SD)^e^		7.5 (12)	8.1 (13)	6.9 (11)

^a^Not applicable.

^b^Missing data for 6 (10%) participants.

^c^Missing data for 11 (19%) participants.

^d^For example, eye disease, neuropathy, kidney disease, or sexual dysfunction.

^e^Missing data for 1 (2%) participant.

### Feasibility of Study Procedures

**Figure 2:**
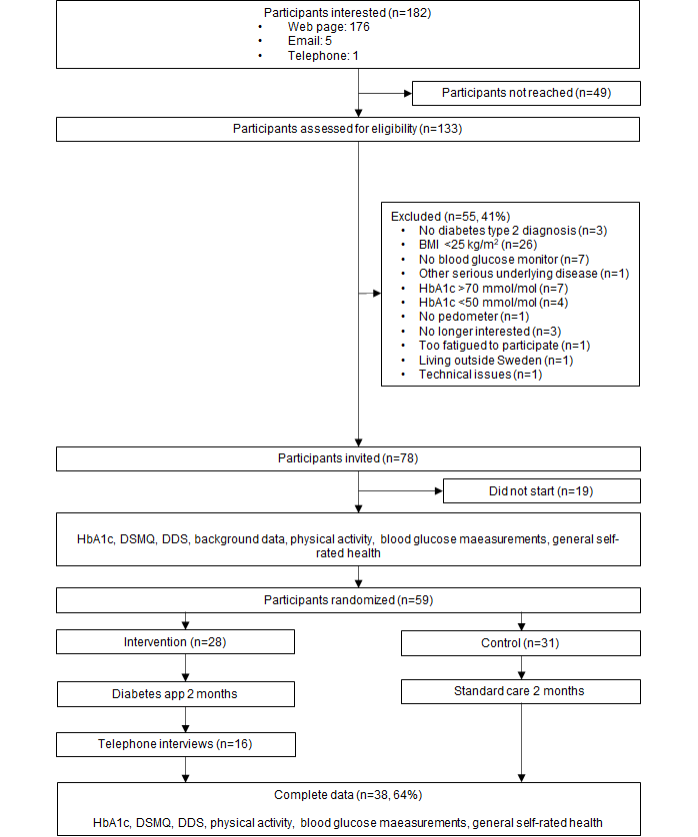
Recruitment flow. DDS: Diabetes Distress Scale; DSMQ: Diabetes Self-Management Questionnaire; HbA_1c_: hemoglobin A_1c_.

Table 2 summarizes the progression criteria fulfilment. There were 182 people that reported interest in participating in the study; of this group, the majority (176/182, 96.7%) registered their interest on a web page. That met progression criterion 1 (n≥60) by a good margin. Of the 182 people interested, 133 were reached and assessed for eligibility. However, a considerable proportion of the participants who registered their interest were ineligible or unable to be included in the study; hence, progression criterion 2 (50% inclusion rate) was not met. The main reason for exclusion at this stage was a BMI <25 kg/m^2^ (n=26). In total, 55 people were excluded. Of those eligible, 19 people never logged into the portal even after being reminded. Finally, 59 (76%) of the 78 eligible participants were randomized in the study (treatment: n=28; control: n=31). For the complete recruitment flow, see Figure 2.

**Table 2:** Summary of the progression criteria with goals and study values.

Progression criteria	Value	Goal reached
(1) At least 60 people reported interest in participating in the study within 3 months of recruitment.	182 people (in 2 months and 12 days)	Yes
(2) At least 50% of those who reported interest were eligible for inclusion in the study.	43% (78/182)	No
(3) At least 75% of those randomized in the study completed the postmeasurements (ie, had complete and valid data).	64% (38/59) with complete questionnaire data and HbA_1c_ test results 81% (48/59) with complete questionnaire data 70% (41/59) with complete HbA_1c_ test results	Partially
(4) At least 80% of those initially interested and eligible actually started participating.	76% (59/78)	No
(5) At least 50% of those who participated in the intervention sent at least 8 blood glucose measurements during the 2-month intervention (about 1 per week).	11% (3/28; based on the “Number of sent diagnostic data”)	No

Progression criterion 3, at least 75% complete data at follow-up, was met regarding questionnaire data (81%). However, when also considering HbA_1c_ tests, the completeness was 64%; thus, the criterion was not met. At baseline, 5 HbA_1c_ test results were missing. At follow-up, 11 randomized participants did not complete their questionnaires, and there were 18 missing tests (Table 3). For baseline HbA_1c_, 41 manual reminders were sent in total; for the follow-up HbA_1c_, 63 manual reminders were sent. See Table 3 for details.

**Table 3:** Feasibility data (n=59).

Data collected			Results
**Baseline**			
	**Self-reported instruments**		
		Time between inclusion and completion of the baseline instruments (days), mean (SD)	5.7 (6.0)
	**HbA_1c_^a^**		
		Time between being sent the test kit and the test results (days), mean (SD)	15.6 (6.4)
		Manual reminders for HbA_1c_, n	41
**Follow-up**			
	**Self-reported instruments**		
		Participants with complete data, n	48
		Time between the prompt and completion of the follow-up instruments (days), mean (SD)	4.4 (4.1)
		Manual reminders, n	37
	**HbA_1c_**		
		HbA_1c_ test results^b^, n	42
		Time between being sent the test kit and the test results (days), mean (SD)	15.4 (8.7)
		Manual reminders for HbA_1c_, n	63
**Sukaribit user data**			
	**Participant activity**		
		Active participants, n	27
		Number of messages sent per participant, mean (range)	1.0 (1-5)
		Number of messages received from physicians per participant, mean (range)	3.0 (0-6)
		Technical issues reported by participants to the developer, n	4
	**Physician activity**		
		Time spent on all participant responses per week (hours)	2
		Time spent on participant responses per week per participant (minutes)	5
		Technical issues reported by the physician to the developer, n	5

^a^HbA_1c_: hemoglobin A_1c_.

^b^Missing tests + defective tests: n=18.

### Feasibility of the Intervention

Of the 28 participants in the treatment group who completed the study, 27 were active users of the app (ie, they completed 2299 data entries in total [blood glucose value, blood pressure value, and medications] in the app and sent 211 of the entries to the physician at some point). In addition, they sent 28 text messages to the physician (see Table 3).

For the 4 participants who requested technical support while using the app, the reasons for contact included difficulties logging in, issues with iOS graphic data, messages not being sent, or that the app had stopped working altogether.

Considering progression criterion 4, 76% of the eligible people actually started participating in the study. This was slightly lower than the criterion of 80%. Regarding criterion 5, only 11% of the participants sent diagnostic data 8 times in 2 months; thus, this criterion was not met.

### Client Satisfaction and the Physician’s Evaluation

A summary of the interviews is presented in Table 4. The findings show that smartphones with the iOS operating system were the most commonly used among the responding participants (15/20, 75%). Concerning the overall quality of the app, a majority of the participants reported the app was of fair quality, with only a few of their individual needs having been met. The 4 technical issues reported to the developer mainly concerned the iOS version of the app. The physician had technical problems but thought the contact was rewarding when it worked. She also experienced varying activity from the participants (Table 4).

**Table 4:** Participants’ (n=20) answers from the telephone interviews and open-ended questions after completing the intervention, as well as the physician’s (n=1) evaluation.

Questions and categories		Participants, n
**Expectations for the app and study**		
	Want to have contact with a physician or health care professional (feedback)	8
	Interest in diabetes apps	6
	Contribute to research	4
	Thinking that a diabetes app is part of the future for diabetes care	3
	Help with more motivation to perform self-care	2
	Want more knowledge	3
	Ability to collect everything in the same place (though it is not working)/facilitate everyday life	2
**Thoughts about the app**		
	Technical problems	12
	Difficult to add their medicine in the list	8
	Technical problems when sending messages/values to the physician	3
	Thought the app was difficult	6
	Did not like the appearance of the app	2
	Easy to navigate	8
	Simple but functioning	2
	Easier to manage more frequent blood glucose monitoring	3
	Easy access to and communication with health care staff	3
	Possible to get feedback on test results from physician	3
	Increased motivation for self-care/increased awareness	3
	Interesting to see how blood glucose is affected by food	1
	Possibility to log data/follow data over time	3
	Good support from the developer	2
**Contact with the physician**		
	Good and relevant replies	9
	Good contact and fast communication	3
	Some sort of miscommunication due to technical issues and maybe a lack of personal knowledge	1
	No/very little communication with the physician	4
**Desired improvements**		
	Wish for an easier app	4
	Improved design	1
	Faster and more communication with caregiver	2
	Direct communication between the app and blood glucose meter	5
	Linked to other health applications	3
	See old values and a graph function (to be able to learn)	4
	Notifications when receiving message overview/table/graph	2
	Wanted bigger text or a computer version	2
	Information/news about diabetes in the app	3
	Be able to send photos	1
	Be able to log physical activities	1
	Be able to set goals	2
	Be able to add notes to values	3
	No function was missing	1
**Overall impression**		
	The application did not improve self-care.	11
	The app improved self-care.	4
	The participants were positive about the concept and think the app should continue to be developed.	3
**The physician’s evaluation**		
	Lots of technical problems (messages, medicine list)	1
	The contact and work were fun when the app worked.	1
	Disadvantage not being their attending physician	1
	The app as a good complement to diabetes care; could consider using it with her own patients	1
	Varying participation of the participants; some very active but others never replied	1
	Room for many improvements	1
	Part of the future	1

### Effect Sizes of Outcome Measures

The effects of treatment on a number of potential outcomes were analyzed based on complete data. No imputations were used. Both the within and between-group Cohen *d* values suggested, at best, small effects. The largest between-group effect size (*d*=0.36) was achieved for the EQ-5D-VAS, and the effect was mainly dependent on the decrease in the control group. In the linear regression analysis, only the number of blood glucose tests per day was significant, indicating 0.57 more tests per day in the intervention group than in the control group (adjusted beta=0.57, 95% CI 0.09-1.06). This effect resulted from a reduction of tests per day in the control group, while the treatment group remained at a stable level. See Table 5.

**Table 5:** Complete case analyses of outcome measures.

Outcome measures	Treatment group				Control group				Between group (post), *d^a^*	Linear regression analyses	
	Baseline, mean (SD)	Post, mean (SD)	n	Within-group, *d*	Baseline, mean (SD)	Post, mean (SD)	n	Within-group, *d*		Adjusted beta^b^	P value
HbA_1c_ (mmol/mol)	50.1 (6.91)	49.3 (7.15)	20	–0.11	55.6 (7.52)	56.3 (7.41)	19	0.08	–0.21	–1.43	.14
Blood glucose tests per day	1.27 (1.62)	1.23 (1.14)	20	–0.03	1.08 (1.62)	0.74 (0.66)	26	–0.30	0.19	0.57	.02
Physical activity per week^c^	3.25 (1.8)	3.15 (1.8)	20	–0.06	3.75 (1.9)	3.71 (2.0)	28	–0.02	–0.03	0.04	.86
Steps per day	4761 (3099)	5407 (3117)	18	0.21	4900 (4371)	5472 (4533)	27	0.13	0.02	188	.88
EQ-5D-VAS (1-100)	55.1 (22.8)	57.8 (25.5)	18	0.11	71.4 (17.3)	67.0 (20.8)	27	–0.23	0.36	3.7	.57
Total DDS^d^	2.45 (0.83)	2.35 (0.76)	20	–0.12	2.00 (0.73)	1.83 (0.70)	28	–0.25	0.09	0.18	.19
Total DSMQ^e^	6.62 (1.34)	6.59 (1.53)	19	–0.02	7.08 (1.05)	7.22 (1.28)	28	0.11	–0.14	–0.16	.51

^a^The posttreatment between-group effect size was adjusted for baseline values.

^b^The difference between groups after treatment was adjusted for age, sex, and baseline values of the respective measure. The control group is the reference.

^c^Number of times, in the last month, the participant performed exercise for more than 30 minutes.

^d^DDS: Diabetes Distress Scale.

^e^DSMQ: Diabetes Self-Management Questionnaire.

## Discussion

In this feasibility study, we explored the prerequisites for conducting a larger study (full-scale RCT) to investigate the effect of the smartphone app Sukaribit on glycemic control. Of the 5 prespecified progression criteria, only 1 was fully met, and 1 was partially met. This indicates that improvements should be considered both regarding study procedures and the intervention before further evaluations. The effect sizes were generally small. Given the low amount of participant activity, this was to be expected.

### Feasibility of Recruitment and Data Collection

Considering the recruitment of participants, the number of responses to the advertisements met and even exceeded that specified in criterion 1. However, the proportion of people who could not be reached or were ineligible to participate due to criterion 2 was slightly higher than ideal. Some alterations to the exclusion criteria were already made during the recruitment phase of the study (ie, including people with an HbA_1c_ <50 mmol/mol or age >65 years). This could potentially have resulted in the inclusion of participants with relatively well-managed diabetes (HbA_1c_ <50 mmol/mol) and participants with less technological experience (age >65 years) The remaining exclusion criterion of a BMI <25 kg/m^2^ resulted in the largest number of exclusions. This was thought to exclude participants who would not likely benefit from the intervention. Another way to facilitate the recruitment process could be to add inclusion or exclusion questions on the study-specific website to better be able to reach the right target group.

Adding to the loss of potential participants in the early recruitment phases, the proportion of eligible and initially willing people who did not finally start participating was also slightly lower than that specified in criterion 2 (76% vs 80%). However, we could relatively easily compensate for these losses in recruitment with a longer and more aggressive recruitment campaign and by reconsidering the arguments for the BMI exclusion criteria.

Although data collection from self-reported questionnaires worked well, meaning that progression criterion 3 was partially met, the collection of HbA_1c_ data through home testing kits did not work well. The first problem was the long administration time. The mean time from sending the kits out until the results were received by the project team was 16 days, with the main delay appearing to be at the participant’s home. There was also a large amount of missing data due to both defective tests and missing tests, even though several manual reminders were sent. Hence, there is a need to make the collection of HbA_1c_ data more reliable and efficient. Previous studies have also reported difficulties using these test kits [26]. Better or additional instructions, more telephone reminders, another test kit brand, or another lab are things to consider. Most likely, the biggest advantage can be gained by improving the participant handling of the test and posting. Other ways to handle this could be to conduct this kind of trial within the health care system so that the blood test is managed by health care professionals and not by the participant. Another thing that we could have done differently is to not have HbA_1c_ as an inclusion criterion. In a full-scale trial, the participant’s glycemic control at the start of the intervention could be a minor issue, since it is the effect of the intervention (the difference) that is measured.

### Feasibility and Acceptability of the Intervention

Not all eligible participants who signed up for the trial started the intervention (progression criterion 4). We do not know the reasons for this; possibly, they just regretted the enrollment. When or if conducting a larger study, the possibility that not all who are accepting of study participation will actually join the study needs to be considered.

A few of the participants in the qualitative evaluation thought that the app improved self-care, but the majority did not think so. Many participants appeared to have been less active than anticipated, especially based on the amount of diagnostic data and messages sent to the physician. This was progression criterion 5, which was not met. Some participants described technical issues that interfered with the use of the app (eg, lack of access to pedometer data in the app as well as difficulties logging medications and viewing summary features). These problems could most often be related to the iOS version. This could have had an impact on user motivation leading to less activity. Participants also suggested improvements in the message function and added features when logging data (eg, in the calendar function, graph) in order to make the app more user-friendly. For the app to be beneficial, it is important that it is used. Previous studies [12] have shown that unsatisfied users will be less active and therefore will not benefit from using this kind of app. Multistep tasks, difficult system navigation, limited functionality, and limited interaction are generally the most common and important usability problems.

To improve user activity, the instructions given to the participants could be improved or routine follow-up telephone calls could be conducted with the participants in the treatment group. The intervention itself could have been more specific, with more guidelines for the participants to enhance their participation, and that might have led to more active self-care. However, this might have been perceived as a bigger effort. Nevertheless, the basic features of the app (ie, self-monitoring and facilitating patient-caregiver communication) appear to be valued by participants. For some, it facilitated a shift in routines toward more frequent blood glucose measurements and a larger understanding of the underlying causes of variations in their blood glucose levels. A feature that may enhance patient engagement is personalized content; for example, individual messaging between the caregiver and user seems to have positive effects in other studies. However, this is something that has not been adequately studied [27].

### Effect Sizes

The effect sizes were small or not existing. Due to the feasibility concerns already raised, it would be premature to calculate a sample size for a full-scale RCT based on these results. If one still would, the only significant result was the number of blood tests, which had an effect size of 0.19. This would result in a necessary sample size of 870 (435 per study group; power=.80, α=.05). Based on the HbA_1c_ results, the required sample size would be close to 1000. One could, based on the almost nonexisting effect sizes, reconsider the choice of self-rated outcome measures. However, with the low activity levels, it is difficult to say if the measures were not sensitive enough or if the intervention did not have a large enough impact.

### Clinical Significance

The results of this study demonstrate the importance of conducting a feasibility trial in order to avoid unnecessary financial as well as study burden for those involved. In order to proceed with a larger clinical trial, a number of problems both in study design and the intervention, as described in the previous sections, need to be addressed. The next step then is to perform a sufficiently powered RCT. If the results are favorable, this will be the first step toward clinical evidence for the intervention, and a new digital treatment helping people to better manage their type 2 diabetes may be available shortly [28].

The participants who signed up for this study were particularly interested in mobile apps; therefore, the results from this study are applicable for patients with type 2 diabetes who want a digital aid. The app could complement standard care and possibly increase empowerment and self-care management. The main advantage of this app is that it enables a new and, maybe, faster way for communication between the person with diabetes and the diabetes nurse or general practitioner. This app, along with other available apps, could be suitable for health care now as well as in future, more digital health care [29].

### Limitations

The smartphone app needed improvements during the trial period. Both the participants and study physician experienced development problems. This probably affected the participants’ experiences with the app. Another possible area of development is of the intervention itself, perhaps with a bigger focus on lifestyle and possibly with other professions involved such as a dietitian, physiotherapist, or diabetes nurse. A possible bias in this study was that the study physician was part of the study team. Since she followed the study protocol and was not involved in the data collection, we believe this issue to be of minor importance. However, an independent physician or diabetes nurse would be preferable. The most preferable option would have been to involve the participants’ own family physician or diabetes nurse, who would have had personal knowledge of the patient. Another bias could be that the participants who signed up for this study were particularly interested in mobile apps. Therefore, the results from this study are applicable for people with type 2 diabetes who want a digital aid and not for the entire population.

### Strengths

A strength of this feasibility study is that the trial was rather large and comprehensive for being a feasibility trial. Another strength is that the app and study methods have been evaluated in several ways with both quantitative and qualitative data, and the evaluation placed a lot of emphasis on the participants’ views. It is important to use different types of methods and validated instruments to get a more comprehensive evaluation of a diabetes app [30].

### Conclusion

Recruitment of participants to a future fully powered study may work with adjustments. The collection of HbA_1c_ using home testing constituted a major problem, and an alternative strategy for this measure is warranted. Finally, the app was not used by participants as intended, and further development is needed. In summary, in order to proceed with a larger randomized study, the app and study procedures need improvement.

## Abbreviations

CONSORT: Consolidated Standards of Reporting Trials
DDS: Diabetes Distress Scale
DSMQ: Diabetes Self-Management Questionnaire
HbA_1c_: hemoglobin A_1c_
RCT: randomized controlled trial
U-CARE: Uppsala University Psychosocial Care Program
